# Bis(propan-2-yl) [(1*S*)-1-(4-fluoro­phen­yl)-1-hydr­oxy-2-nitro­ethyl]phospho­nate

**DOI:** 10.1107/S160053680905260X

**Published:** 2009-12-12

**Authors:** Tanmay Mandal, Sampak Samanta, Grant A. Broker, Cong-Gui Zhao, Edward R. T. Tiekink

**Affiliations:** a Department of Chemistry, University of Texas at San Antonio, One UTSA Circle, San Antonio, Texas 78249-0698, USA; b Department of Chemistry, University of Malaya, 50603 Kuala Lumpur, Malaysia

## Abstract

In the title compound, C_14_H_21_FNO_6_P, a staggered conformation about the central P—C bond occurs, with the oxo and hydroxyl groups occupying diagonally opposite positions. The crystal structure features supra­molecular chains mediated by O—H⋯O hydrogen bonds, which propagate in the *a*-axis direction. A C—H⋯O inter­action consolidates the chains. Disorder was resolved for one of the isopropyl groups with a 0.60 (2):0.40 (2) occupancy ratio for the two components.

## Related literature

For background to the enanti­oselective nitro­aldol reaction of α-ketophospho­nates and nitro­methane and for the synthesis, see: Mandal *et al.* (2007 ▶).
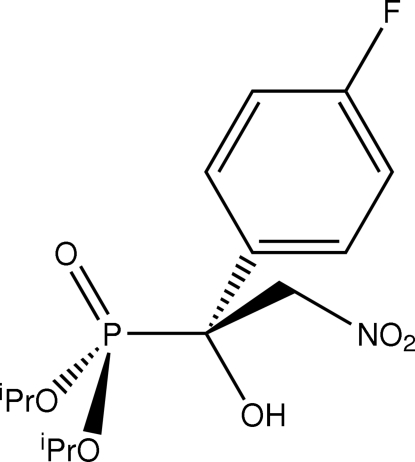


## Experimental

### 

#### Crystal data

C_14_H_21_FNO_6_P
*M* *_r_* = 349.29
Orthorhombic, 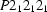

*a* = 5.8267 (12) Å
*b* = 15.931 (3) Å
*c* = 18.273 (4) Å
*V* = 1696.2 (6) Å^3^
*Z* = 4
Mo *K*α radiation
μ = 0.20 mm^−1^
*T* = 173 K
0.31 × 0.15 × 0.06 mm

#### Data collection

Rigaku AFC12/SATURN724 diffractometer
Absorption correction: multi-scan (*ABSCOR*; Higashi, 1995 ▶) *T*_min_ = 0.790, *T*_max_ = 1
6049 measured reflections
3391 independent reflections
3248 reflections with *I* > 2σ(*I*)
*R*_int_ = 0.027
Standard reflections: 0

#### Refinement

*R*[*F* ^2^ > 2σ(*F* ^2^)] = 0.049
*wR*(*F* ^2^) = 0.117
*S* = 1.04
3391 reflections
211 parameters
1 restraint
H-atom parameters constrained
Δρ_max_ = 0.48 e Å^−3^
Δρ_min_ = −0.43 e Å^−3^
Absolute structure: Flack (1983 ▶), 1354 Friedel pairs
Flack parameter: −0.11 (13)

### 

Data collection: *CrystalClear* (Rigaku/MSC, 2005 ▶); cell refinement: *CrystalClear*; data reduction: *CrystalClear*; program(s) used to solve structure: *SHELXS97* (Sheldrick, 2008 ▶); program(s) used to refine structure: *SHELXL97* (Sheldrick, 2008 ▶); molecular graphics: *DIAMOND* (Brandenburg, 2006 ▶); software used to prepare material for publication: *publCIF* (Westrip, 2009 ▶).

## Supplementary Material

Crystal structure: contains datablocks global, I. DOI: 10.1107/S160053680905260X/hb5270sup1.cif

Structure factors: contains datablocks I. DOI: 10.1107/S160053680905260X/hb5270Isup2.hkl

Additional supplementary materials:  crystallographic information; 3D view; checkCIF report

## Figures and Tables

**Table 1:** Hydrogen-bond geometry (Å, °)

*D*—H⋯*A*	*D*—H	H⋯*A*	*D*⋯*A*	*D*—H⋯*A*
O4—H4o⋯O1^i^	0.84	1.94	2.728 (3)	156
C10—H10⋯O5^ii^	0.95	2.52	3.447 (4)	164

Symmetry codes: (i) 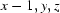; (ii) 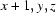.

**Fig. 1.**
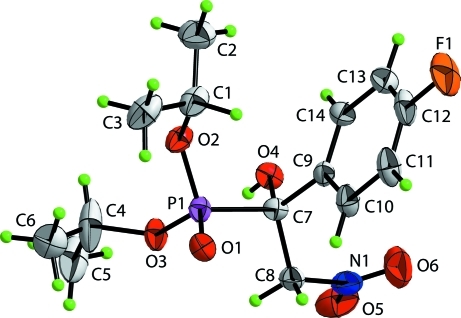
Molecular structure of (I), showing displacement ellipsoids at the 50% probability level. Only the major component of the C6 position is shown (the atom was refined isotropically).

**Fig. 2.**
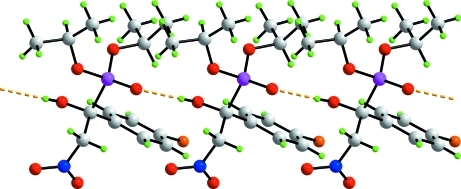
Supramolecular chain along the a axis in (I) mediated by O–H···O (orange dashed lines) hydrogen bonding. Colour scheme: P, pink; F, orange; O, red; N, blue; C, grey; and H, green.

## supplementary crystallographic information

### Comment

In connection with previous studies on the enantioselective nitroaldol reaction of α-ketophosphonates and nitromethane for the synthesis of optically active α-hydroxy-β-nitrophosphonates, the title compound, (I), was investigated. The crystal structure analysis of (I), Fig. 1, shows an *S*-configuration about the C7 atom. When viewed down the P–C7 axis, the molecule has a staggered conformation with the P═O and OH groups being diagonally opposite. The presence of O–H···O hydrogen bonding formed between the hydroxyl-O4—H and O═P atoms leads to the formation of supramolecular chains along [1 0 0], Fig. 2 and Table 1. Stability to these chains of linear topology is afforded by C–H···O contacts, Table 1.

### Experimental

The title compound was prepared as described in the literature (Mandal *et al.*, 2007).

### Refinement

The C-bound H atoms were geometrically placed (C—H = 0.95–1.00 Å) and refined as riding with *U*_iso_(H) = 1.2–1.5*U*_eq_(C). The methyl H-atoms were rotated to fit the electron density. The O–H H atom was located from a difference map and refined with O–H = 0.840±0.001 Å, and with *U*_iso_(H) = 1.5*U*_eq_(O). Disorder is evident in the structure as seen in the anisotropic displacement parameters associated with several residues. However, multiple sites were only resolved for the C6 atom. Two distinct sites were resolved from isotropic refinement of C6/C60 with the major component having a site occupancy factor of 0.60 (2).

### Crystal data

**Table:**

C_14_H_21_FNO_6_P	*F*(000) = 736
*M**_r_* = 349.29	*D*_x_ = 1.368 Mg m^−^^3^
Orthorhombic, *P*2_1_2_1_2_1_	Mo *K*α radiation, λ = 0.71073 Å
Hall symbol: P 2ac 2ab	Cell parameters from 6077 reflections
*a* = 5.8267 (12) Å	θ = 4.2–30.5°
*b* = 15.931 (3) Å	µ = 0.20 mm^−^^1^
*c* = 18.273 (4) Å	*T* = 173 K
*V* = 1696.2 (6) Å^3^	Block, colourless
*Z* = 4	0.31 × 0.15 × 0.06 mm

### Data collection

**Table:**

Rigaku AFC12K/SATURN724 diffractometer	3391 independent reflections
Radiation source: fine-focus sealed tube	3248 reflections with *I* > 2σ(*I*)
graphite	*R*_int_ = 0.027
ω scans	θ_max_ = 26.5°, θ_min_ = 4.2°
Absorption correction: multi-scan (*ABSCOR*; Higashi, 1995)	*h* = −7→5
*T*_min_ = 0.790, *T*_max_ = 1	*k* = −20→13
6049 measured reflections	*l* = −22→18

### Refinement

**Table:**

Refinement on *F*^2^	Secondary atom site location: difference Fourier map
Least-squares matrix: full	Hydrogen site location: inferred from neighbouring sites
*R*[*F*^2^ > 2σ(*F*^2^)] = 0.049	H-atom parameters constrained
*wR*(*F*^2^) = 0.117	*w* = 1/[σ^2^(*F*_o_^2^) + (0.0528*P*)^2^ + 0.8973*P*] where *P* = (*F*_o_^2^ + 2*F*_c_^2^)/3
*S* = 1.04	(Δ/σ)_max_ = 0.001
3391 reflections	Δρ_max_ = 0.48 e Å^−^^3^
211 parameters	Δρ_min_ = −0.43 e Å^−^^3^
1 restraint	Absolute structure: Flack (1983), 1354 Friedel pairs
Primary atom site location: structure-invariant direct methods	Flack parameter: −0.11 (13)

### Special details

**Table:**

Geometry. All e.s.d.'s (except the e.s.d. in the dihedral angle between two l.s. planes) are estimated using the full covariance matrix. The cell e.s.d.'s are taken into account individually in the estimation of e.s.d.'s in distances, angles and torsion angles; correlations between e.s.d.'s in cell parameters are only used when they are defined by crystal symmetry. An approximate (isotropic) treatment of cell e.s.d.'s is used for estimating e.s.d.'s involving l.s. planes.
Refinement. Refinement of *F*^2^ against ALL reflections. The weighted *R*-factor *wR* and goodness of fit *S* are based on *F*^2^, conventional *R*-factors *R* are based on *F*, with *F* set to zero for negative *F*^2^. The threshold expression of *F*^2^ > σ(*F*^2^) is used only for calculating *R*-factors(gt) *etc*. and is not relevant to the choice of reflections for refinement. *R*-factors based on *F*^2^ are statistically about twice as large as those based on *F*, and *R*- factors based on ALL data will be even larger.

### Fractional atomic coordinates and isotropic or equivalent isotropic displacement parameters (Å^2^)

**Table:**

	*x*	*y*	*z*	*U*_iso_*/*U*_eq_	Occ. (<1)
P1	0.90429 (11)	0.45039 (4)	0.97285 (3)	0.02753 (16)	
F1	1.1808 (4)	0.75747 (13)	0.76550 (11)	0.0698 (7)	
O1	1.1339 (3)	0.41823 (11)	0.95590 (10)	0.0332 (4)	
O2	0.8946 (3)	0.52834 (11)	1.02505 (10)	0.0351 (4)	
O3	0.7358 (3)	0.38649 (12)	1.00898 (10)	0.0367 (5)	
O4	0.5136 (3)	0.50239 (11)	0.90853 (11)	0.0321 (4)	
H4O	0.4245	0.4648	0.9237	0.048*	
O5	0.3934 (4)	0.36867 (16)	0.78091 (14)	0.0629 (7)	
O6	0.6260 (6)	0.45661 (17)	0.73135 (13)	0.0832 (10)	
N1	0.5743 (5)	0.40835 (15)	0.77945 (14)	0.0468 (6)	
C1	1.0726 (5)	0.59419 (18)	1.02606 (16)	0.0428 (7)	
H1	1.1352	0.6024	0.9756	0.051*	
C2	0.9465 (7)	0.6725 (2)	1.0502 (2)	0.0619 (10)	
H2A	0.8252	0.6856	1.0149	0.093*	
H2B	1.0545	0.7196	1.0529	0.093*	
H2C	0.8782	0.6630	1.0985	0.093*	
C3	1.2628 (6)	0.5689 (2)	1.07709 (18)	0.0539 (9)	
H3A	1.3373	0.5180	1.0585	0.081*	
H3B	1.1992	0.5578	1.1258	0.081*	
H3C	1.3758	0.6143	1.0802	0.081*	
C4	0.7057 (8)	0.3642 (3)	1.08354 (19)	0.0803 (15)	0.60 (2)
H4	0.6489	0.4155	1.1092	0.096*	0.60 (2)
C5	0.5148 (8)	0.3014 (3)	1.0862 (2)	0.0710 (12)	0.60 (2)
H5A	0.3931	0.3183	1.0522	0.107*	0.60 (2)
H5B	0.4528	0.2987	1.1360	0.107*	0.60 (2)
H5C	0.5735	0.2460	1.0721	0.107*	0.60 (2)
C6	0.9077 (14)	0.3372 (7)	1.1220 (5)	0.058 (2)*	0.60 (2)
H6A	1.0168	0.3839	1.1251	0.087*	0.60 (2)
H6B	0.9786	0.2903	1.0957	0.087*	0.60 (2)
H6C	0.8655	0.3191	1.1715	0.087*	0.60 (2)
C40	0.7057 (8)	0.3642 (3)	1.08354 (19)	0.0803 (15)	0.40 (2)
H40	0.6132	0.4143	1.0983	0.096*	0.40 (2)
C50	0.5148 (8)	0.3014 (3)	1.0862 (2)	0.0710 (12)	0.40 (2)
H50A	0.3931	0.3183	1.0522	0.107*	0.40 (2)
H50B	0.4528	0.2987	1.1360	0.107*	0.40 (2)
H50C	0.5735	0.2460	1.0721	0.107*	0.40 (2)
C60	0.8713 (15)	0.3703 (8)	1.1382 (5)	0.041 (3)*	0.40 (2)
H60A	0.9949	0.4075	1.1218	0.062*	0.40 (2)
H60B	0.9342	0.3145	1.1485	0.062*	0.40 (2)
H60C	0.8014	0.3932	1.1827	0.062*	0.40 (2)
C7	0.7379 (4)	0.47723 (15)	0.88976 (14)	0.0258 (5)	
C8	0.7355 (5)	0.39729 (16)	0.84242 (14)	0.0328 (6)	
H8A	0.8920	0.3858	0.8239	0.039*	
H8B	0.6863	0.3488	0.8725	0.039*	
C9	0.8523 (4)	0.55250 (16)	0.85275 (12)	0.0274 (5)	
C10	1.0637 (5)	0.54344 (19)	0.81713 (14)	0.0376 (6)	
H10	1.1327	0.4896	0.8134	0.045*	
C11	1.1721 (5)	0.6126 (2)	0.78744 (17)	0.0464 (8)	
H11	1.3149	0.6069	0.7629	0.056*	
C12	1.0702 (6)	0.68921 (19)	0.79409 (16)	0.0457 (8)	
C13	0.8621 (6)	0.70094 (18)	0.82673 (16)	0.0435 (7)	
H13	0.7933	0.7550	0.8285	0.052*	
C14	0.7536 (5)	0.63162 (16)	0.85730 (15)	0.0344 (6)	
H14	0.6106	0.6385	0.8815	0.041*	

### Atomic displacement parameters (Å^2^)

**Table:**

	*U*^11^	*U*^22^	*U*^33^	*U*^12^	*U*^13^	*U*^23^
P1	0.0271 (3)	0.0296 (3)	0.0259 (3)	−0.0048 (3)	0.0000 (3)	0.0023 (3)
F1	0.0853 (16)	0.0602 (12)	0.0640 (12)	−0.0366 (12)	0.0008 (12)	0.0286 (10)
O1	0.0281 (9)	0.0341 (9)	0.0373 (9)	−0.0007 (8)	−0.0010 (8)	0.0052 (8)
O2	0.0347 (9)	0.0391 (9)	0.0316 (8)	−0.0100 (8)	0.0013 (9)	−0.0067 (8)
O3	0.0377 (10)	0.0416 (10)	0.0307 (9)	−0.0140 (9)	−0.0015 (8)	0.0084 (8)
O4	0.0241 (9)	0.0320 (10)	0.0402 (11)	−0.0023 (8)	0.0052 (8)	0.0023 (9)
O5	0.0469 (13)	0.0661 (15)	0.0756 (16)	0.0048 (13)	−0.0221 (14)	−0.0285 (13)
O6	0.135 (3)	0.0656 (16)	0.0492 (13)	−0.018 (2)	−0.0420 (18)	0.0132 (13)
N1	0.0594 (17)	0.0359 (12)	0.0451 (14)	0.0120 (13)	−0.0185 (14)	−0.0116 (12)
C1	0.0459 (16)	0.0463 (15)	0.0363 (14)	−0.0209 (14)	−0.0003 (15)	−0.0061 (13)
C2	0.076 (3)	0.0427 (17)	0.067 (2)	−0.0038 (18)	−0.015 (2)	−0.0106 (16)
C3	0.0390 (16)	0.076 (2)	0.0469 (17)	−0.0115 (17)	−0.0008 (15)	−0.0171 (17)
C4	0.092 (3)	0.110 (3)	0.0382 (17)	−0.061 (3)	−0.021 (2)	0.032 (2)
C5	0.081 (3)	0.084 (3)	0.0478 (18)	−0.056 (2)	−0.0012 (19)	0.015 (2)
C40	0.092 (3)	0.110 (3)	0.0382 (17)	−0.061 (3)	−0.021 (2)	0.032 (2)
C50	0.081 (3)	0.084 (3)	0.0478 (18)	−0.056 (2)	−0.0012 (19)	0.015 (2)
C7	0.0230 (11)	0.0238 (11)	0.0308 (12)	−0.0007 (9)	0.0024 (10)	−0.0012 (9)
C8	0.0378 (14)	0.0271 (12)	0.0334 (13)	0.0034 (12)	−0.0096 (12)	−0.0024 (10)
C9	0.0310 (13)	0.0290 (12)	0.0223 (10)	−0.0019 (11)	0.0005 (10)	0.0033 (10)
C10	0.0320 (13)	0.0476 (15)	0.0333 (13)	0.0033 (13)	0.0049 (12)	0.0111 (13)
C11	0.0352 (14)	0.062 (2)	0.0421 (15)	−0.0051 (14)	0.0041 (14)	0.0217 (15)
C12	0.0546 (19)	0.0472 (17)	0.0352 (14)	−0.0202 (16)	−0.0075 (15)	0.0172 (13)
C13	0.060 (2)	0.0307 (13)	0.0398 (14)	−0.0088 (14)	−0.0010 (15)	0.0025 (12)
C14	0.0402 (14)	0.0287 (12)	0.0344 (13)	−0.0039 (12)	0.0011 (13)	−0.0005 (11)

### Geometric parameters (Å, °)

**Table:**

P1—O1	1.4656 (19)	C5—H5C	0.9800
P1—O3	1.5609 (19)	C6—H6A	0.9800
P1—O2	1.5670 (19)	C6—H6B	0.9800
P1—C7	1.851 (3)	C6—H6C	0.9800
F1—C12	1.368 (3)	C40—C60	1.392 (9)
O2—C1	1.476 (3)	C40—C50	1.496 (5)
O3—C40	1.419 (4)	C40—H40	1.0000
O3—C4	1.419 (4)	C50—H50A	0.9800
O4—C7	1.409 (3)	C50—H50B	0.9800
O4—H4O	0.8401	C50—H50C	0.9800
O5—N1	1.229 (4)	C60—H60A	0.9800
O6—N1	1.206 (4)	C60—H60B	0.9800
N1—C8	1.496 (4)	C60—H60C	0.9800
C1—C3	1.503 (5)	C7—C9	1.529 (3)
C1—C2	1.514 (5)	C7—C8	1.540 (3)
C1—H1	1.0000	C8—H8A	0.9900
C2—H2A	0.9800	C8—H8B	0.9900
C2—H2B	0.9800	C9—C14	1.388 (4)
C2—H2C	0.9800	C9—C10	1.401 (4)
C3—H3A	0.9800	C10—C11	1.382 (4)
C3—H3B	0.9800	C10—H10	0.9500
C3—H3C	0.9800	C11—C12	1.362 (5)
C4—C6	1.436 (8)	C11—H11	0.9500
C4—C5	1.496 (5)	C12—C13	1.364 (5)
C4—H4	1.0000	C13—C14	1.390 (4)
C5—H5A	0.9800	C13—H13	0.9500
C5—H5B	0.9800	C14—H14	0.9500
			
O1—P1—O3	115.82 (11)	C60—C40—O3	125.8 (5)
O1—P1—O2	116.03 (11)	C60—C40—C50	122.6 (4)
O3—P1—O2	103.69 (10)	O3—C40—C50	106.9 (3)
O1—P1—C7	112.66 (11)	C60—C40—H40	97.2
O3—P1—C7	99.68 (11)	O3—C40—H40	97.2
O2—P1—C7	107.29 (11)	C50—C40—H40	97.2
C1—O2—P1	123.06 (17)	C40—C50—H50A	109.5
C40—O3—P1	130.3 (2)	C40—C50—H50B	109.5
C4—O3—P1	130.3 (2)	H50A—C50—H50B	109.5
C7—O4—H4O	116.8	C40—C50—H50C	109.5
O6—N1—O5	123.9 (3)	H50A—C50—H50C	109.5
O6—N1—C8	118.6 (3)	H50B—C50—H50C	109.5
O5—N1—C8	117.5 (3)	C40—C60—H60A	109.5
O2—C1—C3	109.6 (2)	C40—C60—H60B	109.5
O2—C1—C2	104.4 (3)	H60A—C60—H60B	109.5
C3—C1—C2	113.5 (3)	C40—C60—H60C	109.5
O2—C1—H1	109.8	H60A—C60—H60C	109.5
C3—C1—H1	109.8	H60B—C60—H60C	109.5
C2—C1—H1	109.8	O4—C7—C9	106.77 (19)
C1—C2—H2A	109.5	O4—C7—C8	111.3 (2)
C1—C2—H2B	109.5	C9—C7—C8	113.8 (2)
H2A—C2—H2B	109.5	O4—C7—P1	110.58 (17)
C1—C2—H2C	109.5	C9—C7—P1	108.40 (16)
H2A—C2—H2C	109.5	C8—C7—P1	105.94 (16)
H2B—C2—H2C	109.5	N1—C8—C7	109.9 (2)
C1—C3—H3A	109.5	N1—C8—H8A	109.7
C1—C3—H3B	109.5	C7—C8—H8A	109.7
H3A—C3—H3B	109.5	N1—C8—H8B	109.7
C1—C3—H3C	109.5	C7—C8—H8B	109.7
H3A—C3—H3C	109.5	H8A—C8—H8B	108.2
H3B—C3—H3C	109.5	C14—C9—C10	119.1 (2)
O3—C4—C6	116.3 (5)	C14—C9—C7	120.3 (2)
O3—C4—C5	106.9 (3)	C10—C9—C7	120.5 (2)
C6—C4—C5	113.2 (4)	C11—C10—C9	120.2 (3)
O3—C4—H4	106.6	C11—C10—H10	119.9
C6—C4—H4	106.6	C9—C10—H10	119.9
C5—C4—H4	106.6	C12—C11—C10	118.7 (3)
C4—C5—H5A	109.5	C12—C11—H11	120.6
C4—C5—H5B	109.5	C10—C11—H11	120.6
H5A—C5—H5B	109.5	C11—C12—C13	123.3 (3)
C4—C5—H5C	109.5	C11—C12—F1	118.2 (3)
H5A—C5—H5C	109.5	C13—C12—F1	118.5 (3)
H5B—C5—H5C	109.5	C12—C13—C14	118.1 (3)
C4—C6—H6A	109.5	C12—C13—H13	120.9
C4—C6—H6B	109.5	C14—C13—H13	120.9
H6A—C6—H6B	109.5	C9—C14—C13	120.6 (3)
C4—C6—H6C	109.5	C9—C14—H14	119.7
H6A—C6—H6C	109.5	C13—C14—H14	119.7
H6B—C6—H6C	109.5		
			
O1—P1—O2—C1	33.2 (2)	O1—P1—C7—C8	55.3 (2)
O3—P1—O2—C1	161.4 (2)	O3—P1—C7—C8	−68.07 (19)
C7—P1—O2—C1	−93.7 (2)	O2—P1—C7—C8	−175.81 (17)
O1—P1—O3—C40	85.5 (4)	O6—N1—C8—C7	69.9 (4)
O2—P1—O3—C40	−42.8 (4)	O5—N1—C8—C7	−108.5 (3)
C7—P1—O3—C40	−153.4 (4)	O4—C7—C8—N1	50.9 (3)
O1—P1—O3—C4	85.5 (4)	C9—C7—C8—N1	−69.8 (3)
O2—P1—O3—C4	−42.8 (4)	P1—C7—C8—N1	171.2 (2)
C7—P1—O3—C4	−153.4 (4)	O4—C7—C9—C14	13.7 (3)
P1—O2—C1—C3	−86.8 (3)	C8—C7—C9—C14	137.0 (2)
P1—O2—C1—C2	151.4 (2)	P1—C7—C9—C14	−105.4 (2)
C40—O3—C4—C6	0(100)	O4—C7—C9—C10	−169.6 (2)
P1—O3—C4—C6	−54.1 (7)	C8—C7—C9—C10	−46.4 (3)
C40—O3—C4—C5	0(8)	P1—C7—C9—C10	71.2 (3)
P1—O3—C4—C5	178.4 (3)	C14—C9—C10—C11	0.4 (4)
C4—O3—C40—C60	0(100)	C7—C9—C10—C11	−176.3 (3)
P1—O3—C40—C60	−25.6 (9)	C9—C10—C11—C12	0.5 (4)
C4—O3—C40—C50	0(8)	C10—C11—C12—C13	−2.1 (5)
P1—O3—C40—C50	178.4 (3)	C10—C11—C12—F1	179.0 (3)
O1—P1—C7—O4	176.02 (15)	C11—C12—C13—C14	2.7 (5)
O3—P1—C7—O4	52.65 (17)	F1—C12—C13—C14	−178.4 (3)
O2—P1—C7—O4	−55.09 (18)	C10—C9—C14—C13	0.3 (4)
O1—P1—C7—C9	−67.26 (19)	C7—C9—C14—C13	177.0 (2)
O3—P1—C7—C9	169.37 (17)	C12—C13—C14—C9	−1.8 (4)
O2—P1—C7—C9	61.64 (19)		

### Hydrogen-bond geometry (Å, °)

**Table:**

*D*—H···*A*	*D*—H	H···*A*	*D*···*A*	*D*—H···*A*
O4—H4o···O1^i^	0.84	1.94	2.728 (3)	156
C10—H10···O5^ii^	0.95	2.52	3.447 (4)	164

Symmetry codes: (i) *x*−1, *y*, *z*; (ii) *x*+1, *y*, *z*.
